# “The Fact That He Was a Police Officer Was Probably My Number 1 Challenge”: Victim-Survivor Experiences of Officer-Involved Domestic Violence in Australia

**DOI:** 10.1177/10778012251319761

**Published:** 2025-02-20

**Authors:** Ellen Reeves, Kate Fitz-Gibbon, Silke Meyer, Sandra Walklate

**Affiliations:** 1 Department of Sociology, Social Policy and Criminology, 4591 University of Liverpool, Liverpool, UK of Great Britain and Northern Ireland; 2 2541 Monash University, Melbourne, Victoria, Australia; 3 5723 Griffith University, Nathan, Queensland, Australia

**Keywords:** officer-involved domestic violence, intimate partner violence, policing, domestic abuse, perpetrator accountability, police accountability

## Abstract

Officer-involved domestic violence (OIDV) is an underexplored phenomena in Australia and internationally. While in recent years some Australian states have adopted OIDV-specific policies, there remains little research which examines the experiences of victim-survivors of OIDV. This article addresses that gap through an examination of the experiences of 17 OIDV victim-survivors. The findings contribute new insights into how OIDV impacts risk; barriers to help-seeking; experiences of reporting OIDV to the police; the risks associated with information sharing; and perpetrator and organizational accountability. These findings underscore the importance of further shaping policy to address OIDV in Australia, while highlighting the necessity for continued research in this area internationally.

## Introduction

Until the early 1990s, with the exception of Cain's, 1973 U.K. study (see Cain, 1973, p. 166), the issue of officer-involved domestic violence (OIDV) was largely unexplored and unacknowledged. Since that time an almost exclusively U.S.-based body of literature has emerged on OIDV. Very little was known about its occurrence in other jurisdictions until more recent work emerging from England and Wales (see Brennan et al., 2023; Casey Review, 2023; Couto et al., 2023; Women's Centre for Justice, 2020). In Australia, empirical research examining OIDV remains virtually nonexistent, despite OIDV gaining increasing traction in the media (Baker & Gladstone, 2023; Gleeson, 2020, 2021). Nevertheless, some Australian states have adopted specific OIDV policies and processes in recent years (Tasmania Police, 2022; Victoria Police Sexual Offences & Family Violence Unit, 2021), prompted by government inquiries (Royal Commission into Family Violence, 2016), victim-survivor-led campaigns, and alarming police data obtained by journalists via Freedom of Information requests (Corbett, 2015; Gleeson, 2020, 2021). Further, and consistent with the lack of an international body of work on OIDV, there is a significant dearth of research examining the perspective and experiences of victim-survivors of OIDV (cf. Mulvihill & Sweeting, 2024).

This article addresses this evidence gap through an exploratory examination of the experiences of 17 OIDV victim-survivors in Australia, drawing on two recent studies conducted by the authors. The first was a study of Australians’ experiences of coercive control in a DV context, wherein we surveyed 1,261 victim-survivors and conducted 170 follow-up interviews. In this study, 11 survey participants cited experiences of OIDV and three of these participated in follow-up interviews. The second study was a victim-survivor advocate consultation providing lived experience insight to inform the Australian Commonwealth Government's National Plan to End Violence Against Women and Children 2022–2032. In this study, 80 victim-survivor advocates were interviewed of which six shared their experiences of OIDV. Neither study was aimed at the detection of OIDV and participants did not necessarily discuss the professional identity of their abusers. The prevalence observed of OIDV across both samples is therefore unlikely to be reflective of the extent of OIDV affecting victim-survivors in Australia or elsewhere. For this reason, we do not make claims here of the broader generalizability of this sample. However, all OIDV victim-survivors across both studies sought formal help for OIDV and experienced complex challenges in reporting their experience and securing a response to support their safety needs. This echoes some of the findings of the Women's Centre for Justice (2020). Hence this work provides unique insights into the significant challenges faced by these OIDV victim-survivors when they tried to report police perpetrators to the very institution to which they belong and work.

This article is structured into four sections. It begins with a summary of existing literature—which, as noted above, is limited and predominantly U.S.-based. Part two provides an overview of our research design. Drawing on data from victim-survivor experiences of OIDV, in the third section, we examine their experiences within the themes of how OIDV impacts their sense of risk; barriers to help-seeking; experiencing of reporting OIDV to the police; safety and information sharing; and perpetrator and police organizational accountability. The final part of this article considers the implications of our findings for policy and practice and emphasizes the need for further research on OIDV in Australia and elsewhere internationally.

## Background

Prevalence rates of OIDV vary significantly across existing studies ranging from 4.8% to 40% (Mennicke & Ropes, 2016). There are notable challenges to measuring the occurrence of OIDV such as the reliance on officer self-reporting, a focus on only physical violence in many studies, and the timeframes used (e.g., determining whether officers are reporting abuse committed before or after they became police officers; Goodmark, 2015; Mennicke & Ropes, 2016; Zavala et al., 2015). However, research generally agrees that DV is more prevalent in police officer families than “civilian” families (Ammons, 2005; French & Fletcher, 2023), though the recent work of Brennan et al. (2023) suggests prevalence rates in England and Wales are similar across both populations.

Existing research has largely focused on why police officers may commit DV, looking at social learning theory and childhood abuse (Prost et al., 2020), general strain theory (Zavala et al., 2015), occupational stress (Edwards, 2006; Gibson et al., 2001; Oehme et al., 2011) and police subculture (Blumenstein et al., 2012; Goodmark, 2015). The latter focus is equally critical to understanding victim-survivor experiences, as police subculture and institutional power are dominant themes in OIDV victim-survivor help-seeking narratives (Mulvihill & Sweeting, 2024). Johnson et al. (2005, p. 4) identify “isolation and police solidarity [as] perhaps the most salient aspects of police subculture” which “creates an ‘us versus them’ attitude.” Isolation may exacerbate occupational stress, create work/home spillover and reinforce the “code of silence” (Blumenstein et al., 2012; Johnson, 2010). Authoritarianism is also a key aspect of traditional police subculture, and a number of researchers have suggested that police institutional power—and the inherent need (and often requirement) of police to be in control—is linked to DV perpetration (Casey Review, 2023; Johnson et al., 2005; Saunders et al., 2016; Women's Centre for Justice, 2020).

Importantly, in some jurisdictions (including Australia) police officers have ready access to weapons which, alongside their in-depth knowledge of the criminal legal system, can be used to prevent victim-survivors from reporting the violence experienced or to facilitate the use of the system against them if they do (Ammons, 2005; Goodmark, 2015; Mennicke & Ropes, 2016; Women's Centre for Justice, 2020). They may also have access to confidential records and personal information, limiting a victim-survivor's ability to covertly seek help (Mazzola, 2014). Victim-survivors may be reluctant to report their partner's abuse in smaller communities where the responding police officer is located and may be an immediate colleague of the abuser and personally known to the victim-survivor (Stone, 2000). Indeed, an aspect of police subculture, is police *families*, whereby spouses of police officers are expected to abide by the “code of silence” hence victim-survivors might be prevented from reporting due to fears of exclusion from their community in addition to the repercussions if their partner is excluded from the community (Johnson, 2010; see also Cain, 1973). Further, Goodmark (2015) suggests victim-survivors of OIDV may feel restricted in their help-seeking due to the presumed prior contacts their partner may have both within and outside of the criminal legal process (on this, see also Casey Review, 2023; Mulvihill & Sweeting, 2024; Women's Centre for Justice, 2020). Barriers such as these can be compounded by police solidarity, whereby fellow officers are often reluctant or unwilling to report or charge their colleagues for DV offenses (Ammons, 2005; Edwards, 2006; Mazzola, 2014; Saunders et al., 2016). Indeed, in many countries, allegations of OIDV are dealt with internally by the police, and investigations frequently amount to no action being taken against abusive officers (Erwin et al., 2005; Gleeson, 2020; Stinson & Liederbach, 2013).

In 1999, the International Association of Chiefs of Police developed a model policy for police forces in relation to OIDV generally considered to be the gold standard internationally (see, inter alia, Royal Commission into Family Violence, 2016). However, the onus is on individual police departments to adopt, shape, and enforce their own OIDV policies and research reveals inconsistency in the willingness to do so (Cheema, 2016; Mazzola, 2014). Police officers continue to “protect their own” and due to the internal nature of the policies, accountability can be minimal (Ammons, 2005, p. 31), thereby limiting the options available to victim-survivors to seek help and recourse if an unsatisfactory police response is received. The importance of mandated policies and accountability have been strongly emphasized by researchers (Avila, 2015; Cheema, 2016; Goodmark, 2015; Mennicke & Ropes, 2016). However, in recognition of the shortcomings of internal policies, focus has also been given to the benefits of preventative measures, such as screening recruits, providing counseling for police officers, awareness campaigns, and specialist training (Ammons, 2005; Cheema, 2016; Johnson, 2010; Mazzola, 2014; Mulvihill & Sweeting, 2024; Oehme et al., 2012; Russell & Pappas, 2018; Zavala et al., 2015).

Until recently, Australian state and territory police forces have made minimal effort to address OIDV—with few even acknowledging its existence. Generally, OIDV policy in Australian states and territories has been to treat OIDV the same as any other form of DV and, where appropriate, to refer matters to internal Professional/Ethical Standards Commands—a response that has been criticized for its lack of independent oversight (Gleeson, 2021). Further, while most Australian states and territories have separate police anticorruption bodies (which vary in their independence from police), they rarely report on OIDV and face significant funding constraints. Two Australian states—Victoria and Tasmania—have recently introduced more concerted policies and processes. In 2021 Victoria Police established an appendage to the Professional Standards Command, the Sexual Offences and Family Violence Unit, to investigate allegations of sexual offenses and family violence against Victoria Police employees where the offending has been categorized as high-risk (Victoria Police Sexual Offences & Family Violence Unit, 2021). The unit's purpose is to oversee regional investigations and victim-survivors can make a direct complaint to the unit. Tasmania has introduced a similar model under their Family and Sexual Violence Involving Police Policy (Tasmania Police, 2022). While also sitting within the Professional Standards Command, the Tasmanian model features an Independent Review Committee, which consists of a range of stakeholders including police, a victim-survivor representative, a legal service representative, and a Safe Families Coordination Unit representative. While not a decision-making body, the committee's purpose is to review decisions made by police and to provide independent oversight.

Data collection for our two studies took place immediately prior to the introduction of these two models and given their recency it is too soon to examine their impact in practice. However, the lived experience perspective on OIDV reported in this article provides some unique and exploratory insights into why specific OIDV policies have value, offers some understanding of the impact of OIDV, and provides important insights to inform what further reforms may be needed to address OIDV. These issues are discussed more fully in the analysis that follows.

## Methodology

This article draws on data from two studies conducted by the authors: a national study of Australian's experiences of coercive control and views on its criminalization^i^; and a national consultation of Australian domestic, family, and sexual violence victim-survivor advocates’ views on the then forthcoming Federal Government's National Plan to End Violence against Women and Children (2022–2032).^ii^ As noted earlier, neither of these studies sought to specifically examine OIDV, meaning that while this comprises a unique and valuable set of exploratory data, it does not purport to capture the full scope and prevalence of OIDV in the relevant samples. Further, the implications of drawing from two different studies (each of which are detailed individually below) should also be acknowledged. The two studies were guided by different research questions, objectives and methods, and thus the context of knowledge production and meaning-making varied. Nevertheless, both studies were orientated toward understanding experiences of, and institutional responses to. DV and thus within this focus the data collected provides key insights into the specific issue of OIDV.

### Study A Design

Study A was conducted from May to November 2021 and drew data from two sources. The first was an online survey open to all Australians aged 18 years and older who had experienced coercive control in a DV context. The survey garnered 1,261 responses. The second data source within this study was 170 follow-up qualitative interviews with survey participants. The survey was advertised widely on social media, via relevant sector newsletters, and by key DV specialist services. Both the survey and the interviews asked participants open-ended questions about their experiences of coercive control; help-seeking experiences; and views on the criminalization of coercive control. Within this sample, 11 survey respondents identified as victim-survivors of OIDV and three of these participated in a follow-up in-depth interview.

### Study B Design

The second study was commissioned by the Commonwealth Department of Social Services (DSS) to inform the development of the National Plan to End Violence against Women and Children 2022–2032 (DSS, 2022). The purpose of this study was to draw on the views of victim-survivor advocates to inform the development of the plan. This work was conducted from October to November 2021 and involved interviews and focus groups with 80 victim-survivor advocates. As this study was specifically interested in the views of victim-survivor *advocates*, participants were recruited via the existing networks of the research team, key DV specialist services, and via publicly available contact details. We spoke to six victim-survivors of OIDV as part of this project—five of whom were interviewed together in a focus group. In relation to the confidentiality of participants in the focus group, these participants were part of an existing advocacy group and requested to be interviewed together.

### Participants With Experience of OIDV

Bringing the data from these two studies together, this article draws specifically on the experiences of 17 victim-survivors of OIDV—all of whom were women. Individual demographics are not available for the National Plan study (Study B), as demographics were recorded anonymously and not in association with specific interviews/transcripts. The authors recognize that this is a key shortcoming as it limits the ability to locate these experiences within broader social contexts and positionality. Demographic data gathered for the coercive control study (Study A, 11 participants in OIDV sample) shows that eight participants were over the age of 41, all were heterosexual, one participant identified as Aboriginal and/or Torres Strait Islander, nine were born in Australia, and all were Australian citizens. Further, seven of the OIDV participants involved in Study A lived in a metropolitan area with the remainder living in a regional (*n* = 3) or rural area (*n* = 1). Participants were highly educated, with five having a Technical and Further Education and/or undergraduate degree, and a further five having a postgraduate degree. Six were employed full time at the time of the interview, and the remainder were employed part time (*n* = 1), casual (*n* = 2), retired (*n* = 1), or were unemployed (*n* = 1). One participant identified as having a disability, and 10 participants had children. While participants’ profession was not specifically captured for either study, one participant identified as a police officer.

Study A also captured the duration of participants’ abusive experiences. Seven of the 11 survey participants were still experiencing coercive control at the time of the survey. Four were no longer experiencing abuse. Of these, two participants said that the abuse stopped 1–3 years ago and 4–6 years ago, respectively. The remaining two participants said the abuse stopped over 7 years ago. Like most participants in Study A, participants in Study B all referred to recent experiences of OIDV. This data highlights that the concerns raised by participants in this study are mostly reflecting on *current* system shortcomings and abuses of power.

### Data Analysis

Analysis of the interview and survey data examined in this article was guided by two exploratory research questions: (a) what are the experiences of victim-survivors of OIDV in Australia and (b) how can these experiences inform police policy reform? To answer these questions, the OIDV data sources from Study A and B were imported into NVivo12 to facilitate qualitative analysis. Using an inductive approach the sources were coded thematically beginning with line-by-line coding and subtheme identification. This was followed by second-level analysis whereby larger themes were identified (Braun & Clarke, 2006). The final four dominant themes are explored in the research findings section below. Throughout the article, and to ensure anonymity of the individuals interviewed, pseudonyms are used when quoting participants and correlate with the specific data source being cited (e.g., Coercive Control Survey A; Coercive Control Interview A; National Plan Focus Group A).

## Research Findings

The research findings are organized into five key themes: how OIDV impacts risk; barriers to help-seeking; reporting OIDV to the police; safety and information sharing; and the importance of perpetrator and organizational accountability. Throughout our analysis we draw heavily on direct quotes from victim-survivors to ensure this article centers their lived experience and perspective.

### How OIDV Impacts Risk

Across the two studies participants often reflected on their own perceptions of their risk throughout their experience of OIDV. Specifically, participants spoke about how their perpetrator being a police officer impacted their level of risk of both further DV and of revictimization when seeking help for DV. As one participant commented about her perpetrator, “he is a state-funded, trained master manipulator.” This manipulation often started from the outset of the relationship, whereby perpetrators were able to draw on the “good image” that came from their profession to present themselves as “safe.” One participant, for example, reflected on how the perpetrator would emphasize his DV-specific policing role as part of his controlling tactics:

(He was) on the board of the local domestic violence center (…) trust me when I tell you that he used that position to outline his credentials as a safe option. You know, ‘I’m really involved in DV.’ I’m safe. I’m on board.’ And then the coercive nature of his behavior crept in. (Coercive Control Interview C)

Indeed, participants recognized that while the pattern of behaviors experienced may be similar to DV committed by civilian perpetrators, it is police officer perpetrators’ unique skills of control, surveillance and investigation, and the resources enabling these skills, which can often exacerbate risk:

(…) you're not dealing with somebody who is just committing offences like a normal Tom, Dick or Harry. They’re doing things that they believe they can get away with or that they know they can get away with (…). Police offenders are smarter than that and they’re looking for these little insidious ways to skirt the system. (National Plan Interview A)

Participants cited a range of resources and knowledge unique to police officer perpetrators. For example, one participant stated “my partner was a cop so I was worried about his access to guns” (Coercive Control Survey F) and another referred to the use of and access to technology within the context of evidencing the breach of an intervention order:

And he came right up to me, like was within 1–2 m, and had his phone up. Appeared to be recording me, can’t be sure he was. (…) So, that's a breach of the (intervention order) in that he is not allowed to keep me under surveillance. And he is not allowed to come within 5 m of me. Anyway, he's been using the recording as evidence that I lied to prove that apparently, he didn’t come within 5 m. I would suggest, if that recording shows that he didn’t come within 5 m of me, that's because he would have stopped the recording at the moment he did. But this has been used as evidence despite nobody actually seeing it. (Coercive Control Interview A)

Interestingly, one participant from the National Plan study was a police officer herself (as was the perpetrator)—the knowledge and resources that she was able to utilize to protect herself offers some insight into the tools at a perpetrator's disposal:

So, even before I went to the police, I had already installed sensor lights on my home, CCTV cameras, a ring video doorbell. I had changed the locks. I had put dowel in all of the window and door sliders so that the windows, etc., couldn’t be forced open. I had sold my car so that he wouldn’t know what vehicle I was driving. Once my next car was identified by him, I changed the number plates. Then I installed a front and rear dash cam in that car. I also installed an application on my phone that is like an emergency SOS, so it has a proforma text message that I had written out in there and it sends my GPS location. If I hit one button it will send it I think to 12 different contacts on my phone (…). (National Plan Interview A)

While this participant was equipped with the knowledge needed to adopt safety measures that would match the perpetrator's own knowledge and skillset, such knowledge is often not available to nonpolice officer victim-survivors.

A less tangible resource cited by several participants across both studies was police “knowledge”—particularly knowledge about DV perpetration and how it is policed. One participant who experienced coercive control from her police officer father-in-law said that he “knows how to make sure that there is no evidence” (Coercive Control Survey K). Other victim-survivors spoke about perpetrators utilizing the fact that nonphysical forms of abuse are underpoliced: “cops who are very clever, they don’t want to leave marks and bruises because they know. It's the coercive (control)” (National Plan Focus Group A). A participant from the National Plan study gave an example of her perpetrator's use of covert nonphysical abuse. She recounted an incident where a parcel of incontinence pads was anonymously sent to her home and while “reporting that to the police means nothing at all,” she detailed the alarming context in which she believed they were sent:

(…) the context is the day that I ended things with my ex-partner and the day that I escaped him, things actually turned physical and he among other things choked me and I was so panicked and I was so distressed and I was just so scared and I don't even know if I did or didn't lose consciousness, but I pissed myself, literally. (National Plan Interview A)

This participant believed the perpetrator sent the package to both humiliate and threaten her. She went on to state:

The context with OIDV compared to just normal civilian DV, I would argue the context is even more important because they know the law, they know how to skirt around it and what reasonable suspicion is and isn’t and how dots are connected. (National Plan Interview A)

Victim-survivors interviewed also described the ways in which police officer perpetrators may also draw on their relationships with other institutions as a resource in their perpetration of DV. This was the case for a participant in our study whose perpetrator threatened to have her committed to a mental health facility:

(…) he was convincing me that I had a mental health issue. He’d get me to a point where I’d be sobbing because he’d tell me everything that was wrong with me and berate me and then say, ‘I can call the police now if I want and get you sectioned and you have to go to (mental health facility) for the night’ (…). (National Plan Focus Group A)

As captured above, police perpetrators having this knowledge and connection with broader formal institutions may deter victim-survivors from reporting the abuse. The range of barriers to help-seeking is explored in more depth below.

### Barriers to Help-Seeking

I feel like I’m in a prison and everything I do is complained about by him to every service I have tried to get help from. (Coercive Control Survey J)

As captured in the quote here, victim-survivors of DV face a myriad of barriers to help-seeking and for victim-survivors of OIDV specifically, these barriers may be intensified. For example, police perpetrators leveraged their profession to prevent the victim-survivor from seeking formal help, reminding them that if they are accused of DV, they may lose their job. Participants who experienced this did not want their perpetrator to be unemployed, and were cognisant of the risk to their safety if something they did or said resulted in their unemployment:

I kept a great deal of what I experienced to myself because I knew if he was charged he would (lose) his job. I was too fearful of the consequences and knew the risk to me would go up should that happen. (Coercive Control Survey G)

The ability of participants to report their victimization was often impeded by their being isolated from friends, family and community. Some participants indicated that they had to move away from family and friends due to their perpetrator's career—which meant there were few people to seek help from, especially when their networks in a new town/city were also mostly police officers and their families:

(…) as soon as we got together, he was wanting to move and he used his work for that. He got a transfer and then all of a sudden we had to move, so I was moved away from family and friends and what-have-you. And I think now I can totally see how strategic that all was. (Coercive Control Interview B)

Being so enmeshed in police culture and communities, some participants also spoke about how inside knowledge of poor police practice and corruption meant that they did not have faith in the police to respond appropriately should they decide to report the abuse:

I knew of corruption when (perpetrator) did his 4 years to be a sergeant in (redacted) and I knew that his two best friends had been kicked out, one for perjury and perverting the cause of justice, the other one for drug dealing, and I knew a lot of stuff. So the first thing I said, and it was the truth, was, ‘I don’t trust you all, I’m not talking to you.’ (National Plan Focus Group A)

This same participant also said her perpetrator “used to bitch about DVs like just how it's that victim's moment of 15 min of fame, a moment of attention.” They reflected that when a victim-survivor has this insight into police attitudes, it is not surprising that they may be even more reluctant to report their own experience of abuse.

Participants also spoke at length about the challenges of experiencing OIDV in regional and rural areas, where local police officers are more likely to be colleagues of the perpetrator. As one participant explained:

When you’re talking (about) what requires improvement in regional areas, I had to report at the police station where he works where everybody knows everybody. It was almost impossible. So the people coming to interview me are his colleagues. It doesn’t work. You can’t trust them, you don’t feel safe, and even the police stations nearby, it's still regional and they still work with each other. (National Plan Focus Group A)

In this scenario, a victim-survivor in a regional or rural area was limited in their ability to seek formal help. Further, attempts to report to the police could result in the perpetrator becoming aware that the victim-survivor is seeking help, threating their reputation among their police colleagues which could in turn result in retaliation placing the victim-survivor at higher risk.

### Reporting OIDV to the Police

For those participants who did report OIDV, fears that the police would protect their own were often actualized. While this type of police culture may be felt particularly strongly in regional and/or rural areas (as described above), it is certainly not limited to these areas as police solidarity goes beyond solidarity to immediate police colleagues and extends to *institutional* solidarity. Participants from a range of geographic locations, including large cities, spoke about reporting their experience of OIDV to the police and police taking active steps to protect the abuser. This sort of back covering and collusion, according to some participants, came from high up in the police force. In some instances, it was obvious the police were reluctant to investigate their own:

(…) eventually, using every connection that I have, I got the name of the local—you know, the area superintendent, and I complained to her. Because I tried to report his stalking to the local police station. The moment I mentioned the name, I was pretty much told to get the fuck out. (Coercive Control Interview C)

In other instances, participants referred to the default credibility offered to a police officer and how senior officers and/or Professional Standards team members tended to always believe the perpetrator:

When you’re a victim of family violence and you’re up against police officers—even my ex himself who is an ex-cop—he is not a current serving member. There is some belief system ingrained that because they’re a cop and they have sworn an oath, that their version of events—if you don’t want to call it lying—is more truthful than the victims. (Coercive Control Interview A)

Relatedly, victim-survivors who reported also said that their perpetrators often tried to actively undermine their credibility—drawing on gendered stereotypes of the “crazy” and unfit mother. As one participant said:

There was this narrative that I was cheating on him, I was cheating on him, I was crazy, I was a negligent mother and I’d leave the kids in the house unattended. All of this stuff was untrue. (National Plan Focus Group A)

Another participant reflected on how far the lies of a police perpetrator can go in a system that favors their narrative:

(…) I took probably 2 years longer to leave than what I wanted to leave. And that delay was because of my fear of him and my awareness that things were going to get worse when I left. Because of his position in the police force. And I knew enough by what I had seen and heard that it was highly likely the police force was going to support him. I clung to hope and I was very naive in the early years that people would do the right thing. And I lived on the hope that—I’ve always been told the truth eventually comes out. Whatever happens initially the truth eventually comes out. But I’m sitting here today 7 years later. He has lied in family court. The truth has never come out. He has lied in the criminal matter. The truth has never come out. He has now lied multiple times in the Magistrates Court. He has played different games with his mental health in the different courts. He has played different games with his version of events with regards to his crime in the different courts. The truth has never come out. (Coercive Control Interview A)

Participants felt that the credibility given to police officers, and the validation of their narratives over the victim-survivors, empowered them to continue the abuse unabated:

I know in my case me going to the police the first time was a massive mistake. The fact that they did nothing about it, they just had a chat to him and he went, ‘No, that didn’t happen’ and then that was it, he just got more and more and more empowered. (National Plan Focus Group A)

The credibility of the perpetrator often transcended police responses, impacting victim-survivors at other points of the justice system. For example, some participants reflected on how the police officer perpetrator had the upper hand in the court room due to existing relationships with court personnel, including magistrates:

(…) the magistrate had a completely false view of the whole situation. She had no understanding of the situation, actually. But add to that, he worked as a police prosecutor in the same court as that magistrate for many years. I believe they are known to each other. (Coercive Control Interview A)

Relatedly, another key concern cited by participants across both studies was the perceived unofficial higher standard of evidence required in civil and criminal DV proceedings against a police officer when compared to a civilian perpetrator—making it harder for victim-survivors of OIDV to prove their victimization. For example, one participant recounted what multiple solicitors told her when she contacted them for advice:

(…) every time I spoke to a solicitor, they’d say, ‘Oh, well. You’ll have such a—you’ll have a far higher threshold to prove anything because he's a police officer, and magistrates don’t like giving orders against police officers because they get made non-operational.’ Now, the threshold should actually be lower for a police officer, not higher (…). (Coercive Control Interview C)

This same participant received support from a senior police officer, who told her that they would be unable to charge the perpetrator with DV offenses, but they were “going to go after him for absolutely everything else we can find.” This experience highlights that even where officers are supportive of victim-survivors, the possible higher threshold for police officers may create additional barriers to securing DV-related charges, prosecution and conviction. Just as it is harder to prove DV against a police officer, it is also easier for police officer perpetrators to convince the legal system that the victim-survivor is a perpetrator themselves. This was experienced by several participants who found themselves listed as respondents on intervention orders or were arrested on DV charges after they had reported the police perpetrator (see also Johnson, 2010; Stone, 2000). As one coercive control survey participant stated:

… my ex is a policeman (…) his friends served me with (intervention orders). It has been biased in his favor from day one. (Coercive Control Survey E)

Indeed, while legal systems abuse is a commonly experienced—and increasingly recognized—behavior experienced by many victim-survivors of DV (Douglas, 2018; Reeves et al., 2023), police perpetrators have unique resources to use the law as a weapon. For example, one victim-survivor reflected on how the perpetrator's knowledge of the system, in addition to his support from the police, resulted in her being charged with assault:

So in my case my ex was the coercive control champion; he’d wake you up all night, he’d break in, he’d destroy property, intimidation. He did do an assault but it wasn’t an assault—it didn’t leave a mark, but then he said that I had dug my fingernails into his hand and that's what I was charged on the basis of. Minor, minor injury that I actually saw him do on the back of a horse float. So I ended up with assault occasioning an actual bodily harm over that. (National Plan Focus Group A)

One victim-survivor reflected on the impact of their own lack of knowledge of the system compared to their perpetrator when they reported the abuse:

I was an idiot, I actually trusted them. I dealt with a couple of the inspectors who I ended up dealing with through work and I trusted them, and when they went, ‘You don’t want him to get in trouble, we’ll just look after it’ I was like, ‘Okay’ because all I wanted was for it to stop. And that was the biggest mistake I could’ve made. No way in a million years did I ever think I would end up being charged. I’m not somebody who has ever, ever done anything that I would ever think I’d get charged about. I never saw it coming, I was blindsided, zero accountability. I just literally think you can’t trust any of them, and you can’t trust the system that sits behind them. (National Plan Focus Group A)

Another described the legal systems abuse as “the ultimate form of coercive control” (Coercive Control Interview B). Such actions by police perpetrators serve to implore on victim-survivors of OIDV that in legal help-seeking the perpetrator will always have the upper-hand.

### Safety and Information Sharing

A key risk associated with OIDV for victim-survivors is the information police perpetrators have access to—when victim-survivors try to seek formal help, their perpetrator is often one step ahead of them. For example, the perpetrator can, via unauthorized access, use the police system to find out if the victim-survivor has reported them to the police, which police they have reported to, what evidence the victim-survivor has provided, and how the case is progressing. This information may also be readily shared with them by their police colleagues. Throughout our interviews, participants cited experiences where police colleagues disclosed their address to the perpetrator. For example, one participant who had obtained an intervention order recounted: (police) “drive him from the hospital where he had tried to commit suicide to try and guilt me back, and straight to the door of my new residence which he was not meant to know where I lived” (Coercive Control Survey H). Another victim-survivor recounted a time when she reported to the police that the perpetrator had “broke into where I was living, destroyed property, hacked into my accounts, assaulted me, stalked me and intimidated me.” The responding officer told her, “if you think he's using the police systems, let me know” (Coercive Control Interview B). In this instance, it can perhaps be viewed positively that the officer was cognisant the perpetrator might be illegally using police systems to commit abuse. However, it is questionable as to why the police did not take it upon themselves to check if this was the case, rather than putting the onus on the victim-survivor to determine if the perpetrator was indeed (mis)using police systems. It is unclear how the victim-survivor in this case would be expected to prove this.

Participants also cited concerns about police officers involved in their case not declaring a conflict of interest due to their close relationship with the perpetrator. In one instance, a victim-survivor stated that one of the police administrative staff involved with her case was in an intimate relationship with the perpetrator and was passing on information about the case. She explained:

I said, ‘Can you please keep my information safe from this person because she will go back and tell (the perpetrator)’ and they were like, ‘Yes, yes, yes’ and they spoke to her, they said, ‘You’ve got to declare any kind of relationship because it's a conflict of interest.’ She ignored that, lied to them, deceived them, started up the relationship with (the perpetrator) again, and it was up to me to provide evidence that they were still in a relationship. (National Plan Focus Group A)

In this instance, the relationship was investigated and when it was discovered the relationship was ongoing, no action was taken against the administrative worker.

Other victim-survivors interviewed also reflected on instances in which their perpetrators had close friends across various levels of policing. For example, one victim-survivor described that her perpetrator had “tentacles everywhere”—including a best friend who worked in the Independent Broad-based Anti-Corruption Commission in Victoria (National Plan Focus Group A). This evidently poses significant risks to victim-survivors’ confidential information. One participant, the one whose perpetrator was in a relationship with a police administrative worker, also reflected on the fact that victim-survivors are often unaware of the police process, including the fact that conflicts of interests should be declared:

The police literally take advantage of the fact that you don’t know the law and you don’t know your rights. So it wasn’t for almost 2 years that I found out that this inspector who is still an inspector should’ve lodged a conflict of interest right at the point where we separated, but for that 2 years she had access to stuff, and I’m pretty confident she's been one of the people accessing information and leaking information to him. I didn’t know. I could’ve told them about her, I could’ve named her if I was told, but you’re just not told stuff. (National Plan Focus Group A)

During the interviews, the recent reforms in Victoria which saw the establishment of an OIDV specific response within Victoria Police overviewed in part one of this article, were discussed by some participants. While this reform approach was generally viewed as a positive step, one participant reflected that the new policy:

…still permits safety and escape plans to be leaked (…) It doesn’t solve any of the problems, doesn’t give you any confidence as a victim. And we’re fighting really hard for privacy protections for victims, so our information doesn’t leak. (National Plan Focus Group A)

As is evident here, if information breaches occur due to personal relationships, then new policies alone may be unable to address this—with the “boys club” mentality prevailing. Another participant offered similar reflections of the potential effectiveness of new Professional Standards, saying: “I don’t think it's entirely impenetrable in terms of information seeping out. But it's a lot better than things being referred to the same area that you and this person work in” (National Plan Interview A). Looking more broadly than the Victorian reforms specifically, one victim-survivor, a police officer herself, reflected on what change is needed to further improve responses to OIDV. Identifying information sharing as a key concern, she stated:

(…) one of the first and foremost most important and crucial things is that the matter is not referred to anybody that that officer knows or has worked with or currently works with for investigation and that it doesn’t even go not just to the same station, but within the same region. (National Plan Interview A)

This reflection is based on her own knowledge of the system and her personal experience of reporting OIDV, where the case was referred to the station where both she and her perpetrator worked—an outcome which is inappropriate and has significant implications for impartiality.

### The Importance of Perpetrator and Organizational Accountability

As is evident throughout the analysis presented in this article, an underpinning concern of victim-survivors interviewed in our studies pertained to the perceived lack of accountability for police perpetrators within their organization. Indeed, from the victim-survivor vantage point police perpetrators of DV were seen to be protected by their immediate colleagues and the larger institution. While some participants were almost entirely dismissed by the police when they reported the abuse, several participants were successful in having the perpetrator charged and/or obtaining an intervention order against them. However, the professional ramifications for the perpetrator in those cases were, for the victim-survivors, often insufficient. As two participants reflected:

My ex-husband is a police officer. Police sought (an intervention order) for my protection and this was granted for 12 months. He has his weapon taken from him, then returned 2 weeks later. (Coercive Control Survey G)

He didn’t get sacked, they let him resign under false claims that he got (posttraumatic stress disorder) from Black Saturday bushfires that he never attended, and now he's on a nice cushy pension for the rest of his life. (National Plan Focus Group A)

Another participant stated that her perpetrator was simply moved to another location. While she was pleased with the implications of this for her own immediate safety, she remained critical of it as a “solution” to the perpetrator's offending behavior:

And it is the age-old response by police for misbehaving police officers, ‘Okay, we’ll just move them somewhere else. We’ll just move them somewhere else.’ It's really disappointing that that is their response. (Coercive Control Interview C)

One of the frustrations described by victim-survivors interviewed was the complexity and lack of transparency in regard to their own case, and in some instances the internal case against the perpetrator. A victim-survivor interviewed reflected on her case being sent to the Law Enforcement Conduct Commission (LECC), a police oversight body in New South Wales, but then ultimately being diverted back to her local police station. She explained:

So I feel like there was a reasonable amount of evidence and yet my complaint to the LECC was still referred back to (local) police station to investigate. And it's a statewide organization, at the very least. If a complaint to LECC has to be handed back to police, why does it end up back at (my local) station? (…) So there's no systematic change as a result of that (…) it still just leaves you feeling so powerless that there is no comeback. (Coercive Control Interview B)

Indeed, several participants spoke about their engagement with bodies charged with keeping police accountable. In regard to the LECC in New South Wales, one victim-survivor reflected:

(…) my understanding is they’re terribly underfunded and also, if there's a matter that's not serious enough for them to pick up and run with, they send you an email saying, ‘Our intention is to refer this back to New South Wales Police for further investigation. Please contact us if you don’t want us to do that.’ Then it's a matter of where do they refer it back to? (National Plan Interview A)

Other victim-survivors also spoke about reporting to Professional Standards, an internal police oversight body operating in most state and territory police forces, and some of the shortcomings of the response received. In particular, the presence of the “boys club” mentality even in these oversight bodies was highly apparent to victim-survivors:

My situation I think was a lot about covering up for an inspector who had not recorded one of my reports about being assaulted and stalked (…), and that inspector now works in Professional Standards and is a really good friend with Deputy Commissioner (redacted). So I think it's a boys club, they cover up for each other. (National Plan Focus Group A)

(Professional Standards) dodgy up the notes, and then when they charge them they give them the member discount. So for choking me unconscious there's a standalone charge in Victoria they could hit him with, they just put that as intentional cause injury assault. They give him a member discount. Much lower charge than they’d otherwise get if they weren’t a member, because they just want them to resign and quietly go so it doesn’t mess up their statistics and they don’t get the media article. (National Plan Focus Group A)

With such responses from the very body established internally to hold police officers to account, victim-survivors’ of OIDV were often left with nowhere else to go with their complaints. At the same time, throughout our interviews victim-survivors frequently expressed frustration that OIDV cases are often not referred to Professional Standards. For this reason, a number of participants were adamant that there needs to be a dedicated stand-alone OIDV unit at each state and territory level to ensure more effective responses to this form of offending. One victim-survivor explained:

I think all of us, we need a dedicated unit to deal with police officer perpetrated domestic violence, it can’t be dealt with by the regions. (…) and we are getting in Victoria a partial specialized unit for OIDV but only for the higher risk cases, not for the 95%. So we in the regions would still have the same issue with the locals investigating our cases. (National Plan Focus Group A)

Participants took care to emphasize the importance of accountability and zero tolerance—as captured by one survey participant: “Until the police can rid its ranks of perpetrators there will be no change” (Coercive Control Survey C). The “change” that this participant refers to is the overall capacity and effectiveness of the police response to DV, which some participants believed is hindered by institutional tolerance of DV by police members:

And that's the thing with OIDV where people think it's just our niche group and we’re just this little fraction of victims, but the fact is our perpetrators are the ones who call in to go to DV calls. So it affects all domestic violence victims (…). It's a bigger issue than just us and I don’t know that people understand that. (National Plan Focus Group A)

So I just feel that the prevalence of abuse within police ranks is actually something that hinders their ability to respond to DV more broadly. (…) How can they respond to DV when you've got whole towns where they're covering for their own abusers?. (Coercive Control Interview C)

These views point to the importance of accountability beyond the individual police perpetrator offender. Emphasizing the importance of organizational accountability and systems change, victim-survivors of OIDV were clear that without wider cultural change internal police processes are unable to adopt effective responses to OIDV.

## Discussion and Conclusion

This article presents one of the first victim-survivor led analyses of the experience of seeking help following OIDV and the adequacy of police responses to OIDV offending in Australia. While existing research has attempted to theorize the reasons why police may engage in DV (see, e.g., Blumenstein et al., 2012; Goodmark, 2015; Prost et al., 2020; Zavala & Melander, 2019; Zavala et al., 2015), this study examines OIDV from the perspective of those who have been victims of it. Echoing international research findings (Ammons, 2005; Casey Review, 2023; Johnson, 2010; Mulvihill & Sweeting, 2024; Women's Centre for Justice, 2020), our analysis underscores the profound impacts of OIDV on victim-survivor safety, access to justice, as well as perpetrator and organizational accountability, highlighting both individual and systemic consequences. Furthermore, by adopting a victim-centered approach to our analysis, this study documents how gendered experiences of DV, (un)safety, and inequality are compounded for victim-survivors of OIDV by virtue of their perpetrator's workplace and the unique power afforded to police officers. The findings presented in this article speak strongly to the importance of listening to women's own understandings of the risk they face (see further Day et al., 2014; Walklate, 2018). This risk stems for the unique power police perpetrators hold in their ability to commit DV (Goodmark, 2015), but also the barriers that present when victim-survivors of OIDV try to seek help (if they do at all). As the victim-survivors’ experiences analyzed throughout this article attest, police have the power to ensure that victim-survivors’ reports of DV are not taken seriously, to turn the system against the victim-survivor, to paint them as the perpetrator, and to uniquely access and use other key systems, such as mental health systems, to pathologize the victim-survivor. This study also revealed the ways in which the onus may be placed on OIDV victim-survivors to hold the perpetrator to account and to secure their own ongoing safety, such as by proving that the perpetrator is illegally using police information systems and/or notifying investigating police of potential conflicts of interest. These unofficial requirements persist even though victim-survivors are unlikely to know about relationships requiring a conflict of interest to be declared. These are both burdensome tasks only effectively achievable by those inside of the police, thus adding a further barrier to help-seeking and safety for victim-survivors of OIDV.

The prevalence of OIDV among Australian police forces (Baker & Gladstone, 2023; Gleeson, 2020) and the institutional failure to address OIDV has important implications for the broader policing of DV. The victim-survivors’ voices captured throughout this article point to the ways in which coercive and controlling behaviors by perpetrators can be dismissed and trivialized, and how DV perpetrators can, in part, capitalize on the fact that coercive control is so poorly policed—knowing what they can and cannot get away with because they are enabled by a system that continues to focus on single incidents of physical violence. Without addressing OIDV within its ranks, victim-survivor faith in the legal system as a help-giving resource is gravely undermined.

Addressing entrenched masculinist police culture common to police services in Australia and internationally has long been identified as a solution to improving policing responses to gender-based violence (Hoyle, 1998). It is critical that this work continues as a system of genuine police accountability is unlikely to be achieved if the “boy's club” mentality persists. The responsibility to change this culture and to disrupt the code of silence lies with the police at an individual and institutional level. Greater awareness and knowledge of the barriers faced by victim-survivors, such as those outlined in this article, may be a good starting point. At the same time, to navigate the complexities of OIDV help-seeking and enhance safety outcomes for victim-survivors there is an urgent need for a paradigm shift toward independent investigation—by taking away the mechanisms that allow police to cover the backs of their colleagues, a message is sent that this practice is not acceptable. While the development of a specific team in Victoria is an important step forward, it operates as an appendage of the police and thus is also vulnerable to manipulation by police perpetrators and the colleagues that seek to protect them—in the same way that the Professional Standards Command was for some participants in the present study. While the Tasmanian model has emphasized independence, its panel is ultimately not a decision-making panel, with final decisions still lying with the police—thus posing the same risks. Given this, the paradigm shift required entails a comprehensive overhaul of investigative processes ensuring independence at all steps of the process. Essential to this approach is the allocation of adequate funding to oversight bodies, offering an alternative investigative avenue for cases involving allegations of OIDV. Critically, victim-survivors’ experiences of OIDV reported throughout this article emphasize the need for independent processes to achieve organizational and perpetrator accountability and prioritize victim-survivor safety—objectives wholly missing from their own experiences of reporting a police perpetrator. These are not unique to the Australian context and signal the need for increased attention to the specific issue of OIDV internationally.

## Notes

i. For further details of this study, see Fitz-Gibbon et al. (2023).

ii. For the findings of this national victim-survivor advocate’s consultation project, see Fitz-Gibbon et al. (2022).
